# Novel Tumor-Targeting Nanoparticles for Cancer Treatment—A Review

**DOI:** 10.3390/ijms23095253

**Published:** 2022-05-08

**Authors:** Adelina-Gabriela Niculescu, Alexandru Mihai Grumezescu

**Affiliations:** 1 Department of Science and Engineering of Oxide Materials and Nanomaterials, Politehnica University of Bucharest, 011061 Bucharest, Romania; adelina.niculescu@upb.ro; 2 Research Institute of the University of Bucharest—ICUB, University of Bucharest, 050657 Bucharest, Romania; 3 Academy of Romanian Scientists, Ilfov No. 3, 050044 Bucharest, Romania

**Keywords:** cancer treatment, cancer management, nanomedicines, controlled drug delivery, novel nanocarriers, tumor-targeting nanoparticles, combined cancer therapies, theranostics

## Abstract

Being one of the leading causes of death and disability worldwide, cancer represents an ongoing interdisciplinary challenge for the scientific community. As currently used treatments may face limitations in terms of both efficiency and adverse effects, continuous research has been directed towards overcoming existing challenges and finding safer specific alternatives. In particular, increasing interest has been gathered around integrating nanotechnology in cancer management and subsequentially developing various tumor-targeting nanoparticles for cancer applications. In this respect, the present paper briefly describes the most used cancer treatments in clinical practice to set a reference framework for recent research findings, further focusing on the novel developments in the field. More specifically, this review elaborates on the top recent studies concerning various nanomaterials (i.e., carbon-based, metal-based, liposomes, cubosomes, lipid-based, polymer-based, micelles, virus-based, exosomes, and cell membrane-coated nanomaterials) that show promising potential in different cancer applications.

## 1. Introduction

Cancer denotes a group of diseases determined by the malignant form of abnormal tissue growth (neoplasm), resulting in cells without a normal morphology and/or function [1,2,3]. These irregular cells can deceive the immune system, proliferate, perform angiogenesis, and even invade other body parts, leading to life-threatening malignancies [4,5]. Thus, prompt and adequate treatment is required to avoid the uncontrolled growth of tumors and the burden they pose on cancer patients. However, cancer treatment is complicated, especially due to the growing resistance of tumor cells to chemotherapeutic agents and unrestrained metastases cascades in invasion, intravasation, circulation, extravasation, and colonization [6]. Non-pharmaceutical treatments can also be employed, including radiation therapy, surgery, hyperthermia, and stem cell therapy, or combinatorial approaches between two or several therapeutic alternatives can be sought [4,7,8]. Nonetheless, each method has disadvantages, ranging from invasiveness to poor drug solubility, short blood circulation of chemotherapeutics, multidrug resistance, nonspecific targeting, and systemic and local off-target side effects [9,10,11,12].

Therefore, despite the broad range of available methods, there is still an ongoing need to develop safer and more efficient therapeutic strategies. Moreover, improving diagnosis and imaging techniques would allow earlier discovery and better monitoring of cancer progression, helping us to understand the course of the disease and treat it accordingly.

Attempting to fill this gap, nanomedicine emerged as a promising interdisciplinary approach for better managing human health, including the search for potent anticancer tools. Particular research interest has been focused on developing tumor-targeting nanoparticles that can release encapsulated or conjugated bioactive agents in response to the tumor microenvironment’s unique chemical and biological conditions. Such nanocarriers can improve the bioavailability of drugs, efficiently accumulate in the tumor site, favor tumor cell uptake, combine therapeutic agents with imaging techniques, and boost antitumor effects [10,12,13,14].

In this context, this paper aims to extensively discuss the recent advancements in the field of tumor-targeted therapeutic approaches, according to the type of used nanomaterials. Specifically, after briefly presenting the main current cancer treatments, this review elaborates on newly developed nanosystems, including carbon-based nanomaterials, metal-based nanomaterials, liposomal formulations, cubosomes, lipid nanoparticles, polymeric nanoparticles, micelles, exosomes, cell membrane-coated nanoplatforms, virus-like and virus-based nanomaterials, that show a remarkable potential for cancer applications.

## 2. Main Current Treatments

Various strategies can be employed in treating cancer, as choosing the proper therapeutic alternative depends on many factors, including the type of cancer and its characteristics, disease localization, prior treatment history, and general health state of the patient. Hence, this section briefly discusses the main current treatment approaches employed in clinical practice to set the reference framework for recent research.

Chemotherapy remains one of the most common treatment options in fighting cancer. Chemotherapy can be used alone or in combinatorial approaches with radiotherapy, surgery, or adjuvant therapies to produce effective antitumor responses [7,8,9,15,16]. More specifically, chemotherapeutic drugs are administered for inhibiting cell proliferation and tumor multiplication, impeding cancer invasion and metastasis occurrence. Nonetheless, the mechanism of action of chemotherapeutic agents is also responsible for their side effects. This is mainly due to the lack of specificity of drugs that cannot distinguish between cancer cells and other rapidly multiplying normal cells (e.g., bone marrow, gastrointestinal tract, hair follicles) [17].

Moreover, the unsatisfactory specificity, poor aqueous solubility, and short blood circulation of anticancer drugs are further reflected in the presence of low concentrations of chemotherapeutics at the tumor site, imposing a need for administering high doses [4,9,10,12,17]. Furthermore, drug efficacy has been observed to decrease over time with the development of chemoresistance [18,19]. Therefore, complementary therapeutic options must be used together with chemotherapy in order to improve its treatment outcomes. One example of frequently paired therapy is radiation therapy. Specifically, radiotherapy can be employed for destroying the cancer cells previously sensitized by chemotherapeutics. However, radiation also affects normal tissues, leading to the appearance of side effects immediately or soon after radiotherapy treatment [20,21]. Therefore, conventional cancer therapies may be accompanied by many off-target adverse effects, including appetite loss, anemia, internal bleeding, fatigue, and hair loss [4,11,12].

In an effort to improve treatment outcomes and reduce adverse side effects, various adjuvant therapies have been researched and recently started to gain popularity (Table 1).

## 3. Tumor-Targeting Nanoparticles for Cancer Management

As none of the clinically available therapeutic options lack disadvantages, there is an imperative need to overcome the challenges and limitations of conventional and adjuvant anticancer therapies. Particularly, extensive knowledge can be gained from better understanding tumor microenvironments, with the goal of designing more specific therapeutic approaches with enhanced targeting ability to cancer cells.

**Figure 1:**
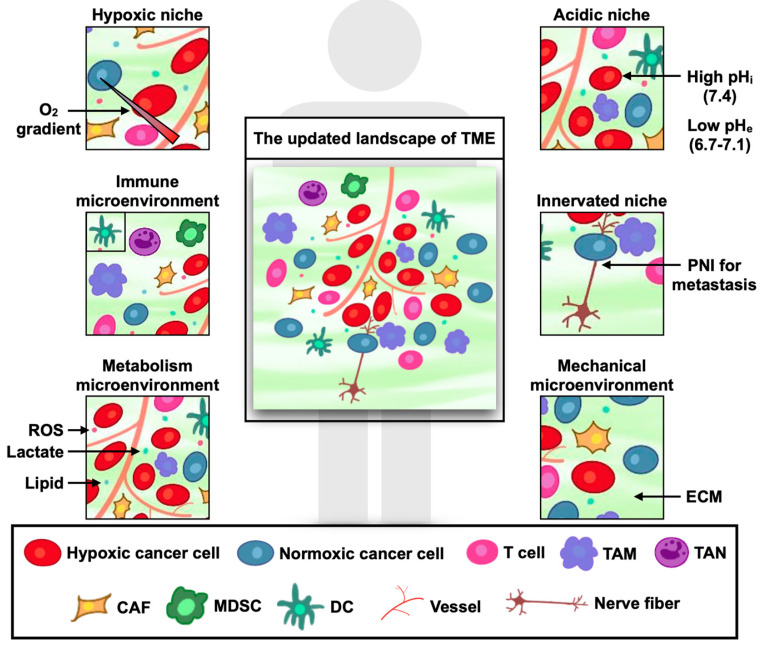
The updated landscape of tumor microenvironment (TME). Reprinted from an open-access source [46]. Abbreviations: CAF—cancer-associated fibroblasts; DC—dendritic cells; ECM—extracellular matrix; MDSC—myeloid-derived suppressor cells; PNI—perineural invasion; ROS—reactive oxygen species TAM—tumor-associated macrophages; TAN—tumor-associated neutrophils.

The tumor microenvironment (TME) is a fundamental component of malignant tissues, often regarded as the “soil” of cancer development. TME comprises unique cellular and noncellular entities that remodel the extracellular matrix (ECM) and control cancer progression [44,45] (Figure 1).

**Figure 2:**
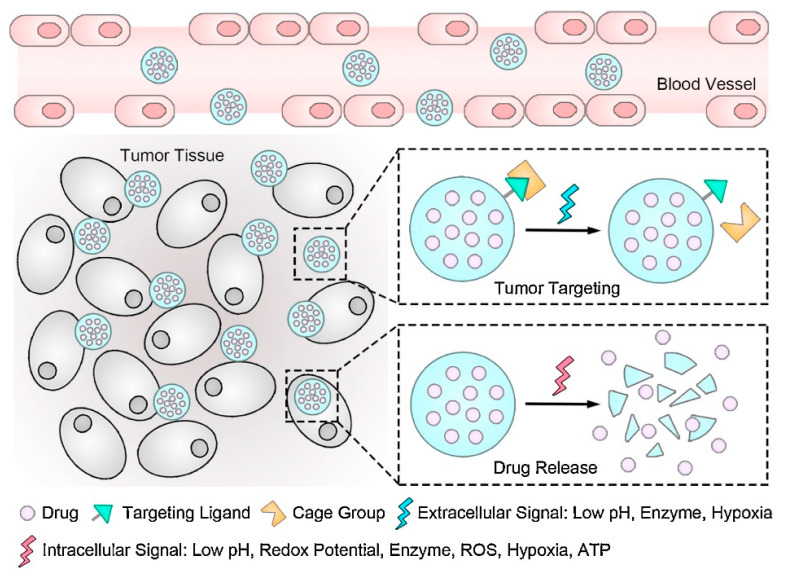
Schematic representation of the role of TME and intracellular signals in tumor targeting and controlled drug release. Reprinted from an open-access source [49]. Abbreviations: ATP—adenosine triphosphate; ROS—reactive oxygen species.

The characteristic hypoxic, hypoglycemic, and acidic conditions of TME [44] represent important triggers for drug release, enabling researchers to create TME-responsive delivery systems. Distinct features, such as irregular vascular structure, dense stroma, and numerous supporting cells (e.g., CAF, TAM) have also been leveraged for efficient cancer diagnosis and treatment. Based on these particular physicochemical parameters, nanobiotechnological modalities can liberate active constituents under various stimuli, counting temperature, pH, redox potential, enzymes, and more [47]. In addition, the overexpression of specific cell surface receptors by tumor cells can be employed for targeting cancer cells via antibodies or smaller molecules with the goal of generating more specific and pronounced antitumor effects and reducing side effects on normal tissues [48] (Figure 2).

**Figure 3:**
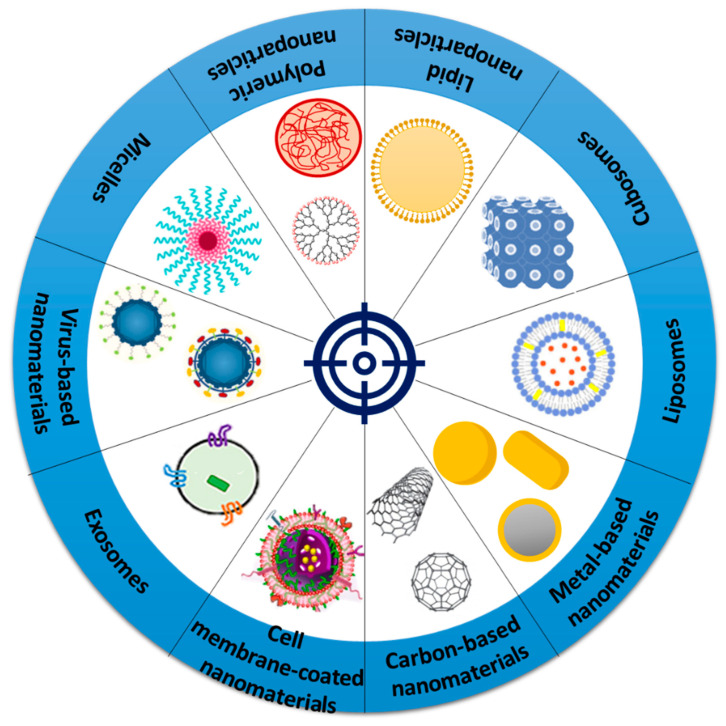
Schematic representation of various nanomaterials researched for tumor-targeting applications.

In this respect, a variety of nanomaterials (Figure 3) have been explored for creating efficient tumor-targeting modalities for cancer treatment.

### 3.1. Carbon-Based Nanomaterials

Given their high surface-to-mass ratio, high loading capacity, and their ability to bind hydrophobic molecules through π–π interactions, carbon-based nanomaterials (CNMs) represent appealing vehicles for the delivery of drugs, genes, and proteins to specific sites. CNMs also exhibit particular optical properties, as the graphitic carbon structure endows them with a strong absorption capability in the near-infrared range (750−1000 nm for the NIR-I window and 1000–1700 nm for the NIR-II window). These properties make CNMs attractive candidates for tumor photothermal therapy, photoacoustic imaging, and deep-tissue fluorescence imaging [44]. Moreover, CNMs are biocompatible, lack immunogenicity, and possess multifunctional surface chemistry, which renders them popular for designing composites with targeting capabilities, remarkably low toxicity, and high pharmaceutical efficiency [50].

**Figure 4:**
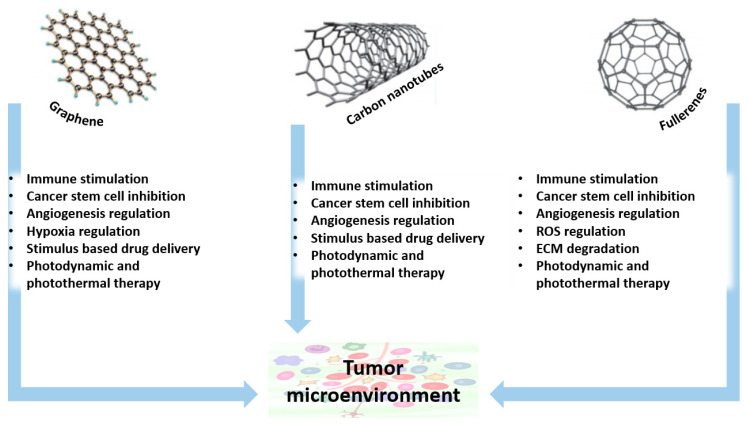
The role of Carbon-based nanomaterials in TME-targeted cancer therapy. Adapted with permission from [44]. Copyright 2018, John Wiley and Sons. Abbreviations: ECM—extracellular matrix; ROS—reactive oxygen species.

Furthermore, CNMs present many dangling bonds on their edges, as well as defective sites that promote catalysis and redox reactions at the interfaces. This quality further recommends the use of CNMs for the controlled, TME-response release of therapeutic molecules in the presence of physical and chemical stimuli. In addition, CNMs have the potential to influence pathways involved in tumor migration and invasion, tumor-associated inflammation, hypoxia, metabolism, and angiogenesis, being promising nanocarriers and nanomedicines for TME-targeted cancer therapy [44,51] (Figure 4).

**Figure 5:**
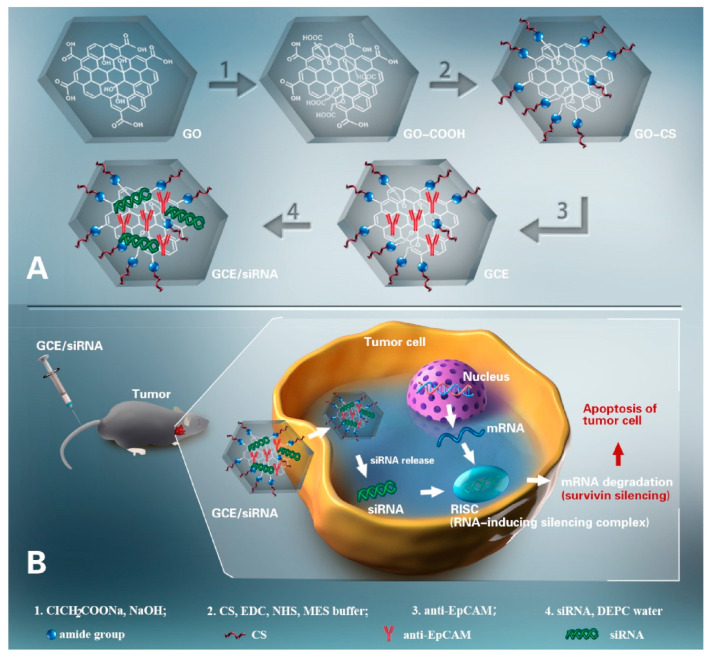
Schematic representation of the delivery system designed by Chen et al. (**A**) Nanosystem fabrication. (**B**) Mechanisms of action. Reprinted from an open-access source [52]. Abbreviations: CS—chitosan; DEPC—diethylpyrocarbonate; EDC—N-(3-dimethylaminopropyl)-N′-ethylcarbodiimide; GCE—GO-CS/anti-EpCAM; GO—graphene oxide; MES—2-(N-morpholino) ethanesulfonic acid; NHS—N-hydroxysuccinimide.

Tackling the advantageous properties of graphene oxide (GO), Chen et al. [52] have modified it with chitosan and a tumor-specific monoclonal antibody (anti-EpCAM) for the delivery of survivin siRNA (Figure 5). The as-designed carrier displayed excellent biosafety and tumor targeting activity through specific binding, superior siRNA loading performance, and an excellent in vitro protection effect for survivin siRNA. These attributes were further reflected in the enhanced effectiveness of the nanosystem, which showed a strong antitumor effect in vitro, associated with efficient antiproliferation and migration and invasion inhibition activity. Moreover, the nanoplatform presented no toxicity for blood and the main organs, proving its biosafety for gene delivery and reconfirming its targeting ability.

Alternatively, Basu et al. [53] have prepared hyaluronic acid-engrafted, metformin-loaded GO nanoparticles. The authors reported anticancer efficacy at a much lower dosage than for the bare drug, with the nanosystem being able to induce apoptosis and inhibit the cell migration of triple-negative breast cancer (TNBC) cells by targeting the miR-10b/PTEN axis via NFkB-p65. The treatment was also noted to inhibit cell migration via a reduction in pFAK/integrinβ1 expressions, inhibiting epithelial-mesenchymal transition, increasing E-cadherin expression, inhibiting mammosphere formation, lowering the expression of stemness markers, and nullifying toxicity in peripheral organs imparted by the tumor.

A different targeted nanocarrier is proposed by Hu et al. [54], who have functionalized graphene with folic acid and gamma-cyclodextrin. This as-designed hybrid construct is capable of hosting pristine C_60_ molecules, creating an effective nanomedicine for cancer treatment. Functionalization with folic acid endowed the nanosystem with a strong tumor-targeting ability, as it could bind to folic acid receptors from the cellular membranes. The authors reported that this nanoplatform promotes cellular uptake and enhances light absorption, being an excellent tool for phototherapy.

Pristine C_60_ fullerenes were also reported to be efficient in complexation with Landomycin A (LA). Bilobrov et al. [55] demonstrated that this nanocomplex had high toxicity for cancer cells, while much lower toxicity was seen for mesenchymal stem cells. Thus, it was concluded that the nanocarrier has a good targeting ability, while the nanocomplex has great potential as an anticancer agent. In contrast, Shi et al. [56] have used fullerenes for the delivery of doxorubicin. The authors created an “off-on” type drug delivery system with precise control by covalently conjugating the anticancer agent to C_60_ nanoaggregates through a ROS-sensitive thioketal linker and adding a hydrophilic shell. The “off” or “on” state of the nanohybrid could be precisely remote-controlled with the aid of a 532 nm laser (at a low power density) with a high spatiotemporal resolution. Thus, the system offers a promising synergistic therapeutic effect, combining the benefits of targeted chemotherapy and phototherapy.

The benefits of carbon nanotubes have also been widely investigated for cancer therapy. Particularly, multiwall carbon nanotubes (MWCNTs) have started to gather increased scientific interest. For instance, Radzi et al. [57] acid-functionalized MWCNTs and used the particles in combination with local hyperthermia therapy. The researchers observed a considerable decrease in cell proliferation compared to the untreated tumor, accompanied by an increase in Hsp70 expression in tumors treated with hyperthermia therapy. The combined treatment also led to an increase in dendritic cell infiltration and maturation and a considerable rise in tumor infiltrated CD8^+^, CD4^+^ T cells, macrophages, and natural killer cells. Therefore, the association of MWCNTs and hyperthermia therapy represents a promising strategy that might be of interest for treating breast cancer.

More recently, Zhou et al. [58] developed a multifunctional nanocarrier for anticancer drugs to be delivered into the specific location of tumor cells. The scientists functionalized MWCNTs with polyethylene glycol (PEG) and used it as a targeting ligand folic acid, bonded with hyperbranched poly-L-lysine and crosslinked via adipic acid. The targeting ligand improved specific delivery to cancer cells, as folic acid covalently conjugated with the hyperbranched polymer, which helped in intracellular drug delivery to folic acid receptor overexpressed tumor cell membranes. Thus, the as-designed, MWCNT-based nanosystem loaded with doxorubicin was tested on human liver cancer cells, leading to increased cytotoxicity and apoptosis in the targeted cells. Singhai et al. [59] also loaded doxorubicin in MWCNTs but used alternative functionalizing agents. The authors employed hyaluronic acid and α-tocopheryl succinate, enhancing cellular placement, increasing cellular uptake, and improving anticancer activity against CD44 receptors overexpressing TNBC cells.

In contrast, Prajapati and colleagues [60] prepared gemcitabine-loaded, hyaluronic-acid-conjugated PEGylated MWCNTs to act as targeted nanomedicines against colon cancer. Encouraging results were obtained, as the system provided a faster release in the acidic medium than at physiological pH, followed by a sustained release pattern. Moreover, in comparison to a free drug, the nanoplatform exhibited significantly less hemolytic toxicity, higher cytotoxicity against the HT-29 colon cancer cell line, considerably reduced tumor volume, and an improved survival rate without noticeable loss in body weight. Thus, the nanosystem represents a potential safe and effective targeted treatment alternative for colon cancer.

### 3.2. Metal-Based Nanomaterials

**Figure 6:**
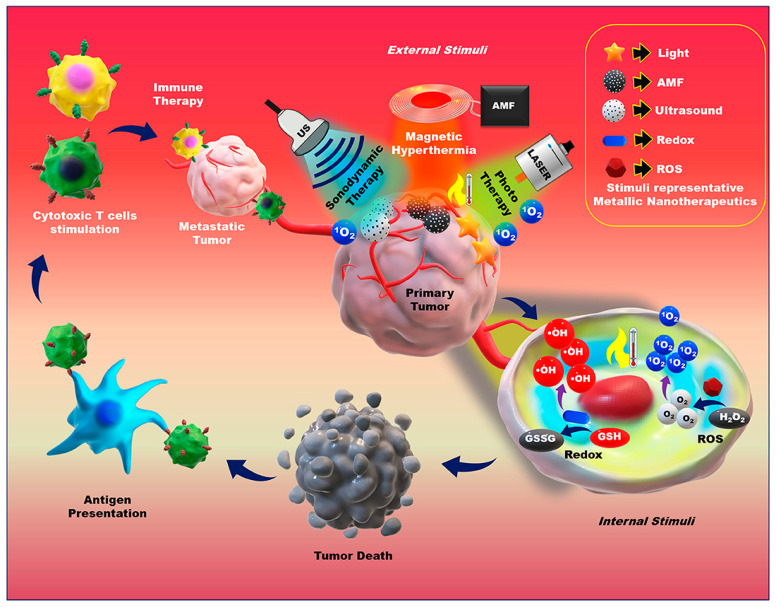
Application of external and internal stimuli-triggered metallic nanotherapeutics for cancer treatment. Reprinted from an open-access source [65]. Abbreviations: AMF—alternative magnetic field; GSH—glutathione; GSSG- glutathione disulfide; ROS—reactive oxygen species; US—ultrasound.

Metallic nanoparticles have also attracted considerable interest for developing tumor-targeted systems due to a series of advantages, including relatively narrow size and shape distribution, long activity period, the potential for dense surface functionalization, and the ability to remodel TME by changing unfavorable conditions into therapeutically accessible ones [61,62,63]. In this context, many in vitro and in vivo studies have investigated the use of metallic nanoparticles against different types of cancers, exploiting their controlled-release ability under a plethora of internal and external stimuli [64,65,66,67] (Figure 6).

For instance, Jin and colleagues [68] fabricated aptamer-modified hollow silica nanoparticles with a pollen structure for the tumor-targeted and pH-responsive delivery of doxorubicin. The authors reported an 87.5% release efficiency at a pH of five, while the special spikes of the nanoconstruct acted as “entry claws”. In this manner, the interaction between cell and drug nanocarriers was enhanced, increasing internalization in target cells, whereas almost no nanoparticles were internalized by healthy cells. In addition, the excellent biocompatibility and cell viability recommend these nanoplatforms for targeted tumor therapy.

Alternatively, Kim et al. [69] developed a US-responsive drug release system. The researchers used doxorubicin-coordinated titanium dioxide nanoparticles for encapsulating polymeric phenyboronic acid. The as-designed nanosystem allowed for a drug release via ROS, generated under US irradiation, showing high tumor accumulation and efficient tumor growth inhibition. The encouraging results obtained with this sonodynamic chemotherapy suggest that the newly developed particles may represent a useful tool in treating cancers that cannot be overcome by a single therapy.

Deng et al. [70] proposed a different smart drug delivery nanosystem. The authors loaded doxorubicin and pirfenidone in zinc oxide/copper sulfide-based nanoparticles, functionalized with β-CD-DMA and PEG-DMA, which activate in the mildly acidic TME. In more detail, the nanosystem became smaller in response to the acidic pH from TME and the charge reversed, ensuring a deep penetration of nanoparticles into cancer cells. Pirfenidone could inhibit CAF activation and enhance tumor penetration, while the residual nanostructure could trigger cascade-amplified ROS generation to induce apoptosis in cancer cells. The photothermal effect augmented antitumor efficacy, leading to a remarkable inhibition of tumor growth and lung metastasis. Through the integration of chemotherapy and phototherapy, this nanosystem provides a promising alternative in treating breast cancer.

An interesting possibility is also found in the use of cetuximab-targeted, [^177^Lu]-gold nanoparticles. Shabbir and colleagues [71] demonstrated that these nanoplatforms have a high affinity for epidermal growth factor receptors, being of a promising utility in the treatment of antibody-resistant colorectal cancer. Moreover, administering these nanoconstructs before radioimmunotherapy has the potential to enhance clinical outcomes.

More recently, Ding et al. [72] designed liquid metal nanoparticles, to which they attached glucose oxidase, mineralized with amorphous calcium carbonate, and decorated with the poly l-aspartic-acid-grafted copolymer PEG-PAsp. The researchers aimed to integrate the synergistic effect of adenosine triphosphate generation inhibition and photothermal therapy for improved tumor therapy. The proposed nanosystem could efficiently oxidize glucose to generate hydrogen peroxide and gluconic acid after the decomposition of calcium carbonate at the tumor site, further stimulating calcium ions to affect mitochondrial function towards a reduction in ATP synthesis.

Metal-based nanomaterials can also improve cancer management through the construction of tools for accurate imaging and diagnosis, which would help initiate treatment at early disease stages. For instance, a study conducted by Liu et al. [66] revealed that ultrasmall Fe@Fe_3_O_4_ nanoparticles modified with 3-(3,4-dihydroxyphenyl)propionic acid (DHCA) and conjugated with F56 peptide have the potential to act as tumor-targeting contrast agents. Nanoparticles of 8 nm displayed optimal *T*_1_–*T*_2_ dual-mode MRI performance and tumor-targeting abilities both in vitro and in vivo. Thus, these particles represent potential candidates for accurate tumor diagnosis. Another research group [67] investigated iron–platinum nanoparticles for imaging purposes. The authors reported that these nanoparticles could achieve a few centimeters deep photoacoustic imaging for the diagnosis of breast tumors, while conjugation with anti-VEGFR endowed them with the ability to target tumor sites. Moreover, the particles were rapidly cleared away from the tumor site and majorly metabolized through the liver, showing low toxicity in the theragnosis of early breast cancer.

### 3.3. Liposomes

**Figure 7:**
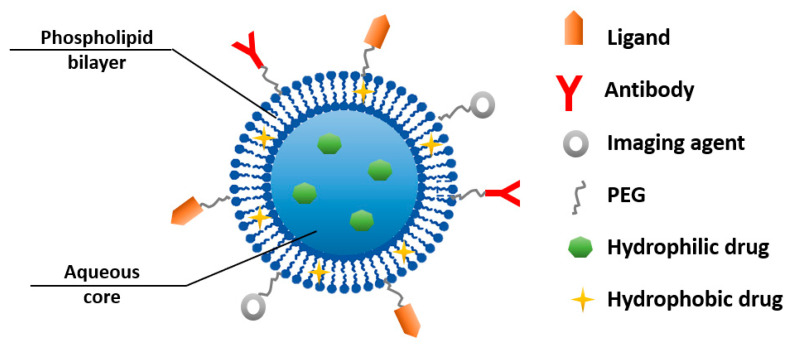
Schematic representation of a multifunctional liposome-based nanoparticle.

Liposomes represent one of the most researched structures for developing performant drug carriers [73]. Their unique construction, with an aqueous core and a surrounding bilayer of phospholipids, allows them to deliver both hydrophilic and hydrophobic drugs [74]. Liposomes offer other beneficial properties as well, including biocompatibility, efficient drug encapsulation, the ability to control their size, and ease of functionalization. However, liposomes are recognized as having a short circulation half-life, which can be overcome by PEGylation. Ease of surface modification also allows for the possibility of creating multifunctional, liposome-based nanoparticles with an enhanced targeting ability with regard to tumor sites [2,48] (Figure 7).

In this respect, Wu et al. [75] designed alendronate and low-molecular-weight-heparin-modified liposomes for the delivery of doxorubicin. Alendronate was used with the dual role of bone targeting and as a antiosteoporosis therapeutic agent, while heparin could improve the blood circulation time of liposomes and exhibit anti-metastasis efficiency. The nanosystem displayed remarkable tumor growth suppression and tumor metastasis inhibition, proving its efficiency against both orthotopic osteosarcoma and breast cancer bone metastasis. Moreover, given the fact that each component of the system is FDA-approved, the as-designed liposomal formulation has considerable potential for practical use.

Cheng et al. [76] also employed liposomes for the delivery of doxorubicin. The authors used ammonium bicarbonate liposomes, functionalized with folate and PEGylated phospholipid with a pH-sensitive imine bond. The nanocarriers possessed an active targeting ability, long circulation time, enhanced cellular uptake, quick intracellular drug release, and promising cytotoxicity in slightly acidic mediums and hyperthermia.

Alternatively, Kim and colleagues [77] loaded liposomes with rituximab through holes made in their surface, which were further plugged using hyaluronic acid grafted with 3-diethylaminopropylamine (DEAP). In TME, due to acidic conditions, the DEAP ionized, leading to the extensive release of the encapsulated drug. Thus, the anticancer agent accumulated at high levels in tumors and bound to the CD20 receptors overexpressed with Burkitt lymphoma Ramos cells, resulting in improved tumor cell ablation.

One study conducted by Kang et al. [78] revealed that large, anionic liposomes administered intraperitoneally could target TAMs for the delivery of resiquimoid. Through this targeted delivery, the drug promoted the activation of M1 macrophages and T cell infiltration, which reduced the percentage of Tregs in TME and increased the efficacy of the PD1 blockade against syngeneic ovarian tumors. Given these promising results, the authors are confident that further optimization of the liposomal nanovehicle has the potential to create a clinically relevant approach for better immunotherapy in ovarian cancer patients.

Wang et al. focused on fabricating smart-response drug delivery systems for targeting L-type amino acid transporter 1(LAT1) and amino acid transporter B^0,+^ (ATB^0,+^) in cancer cells. In 2019, the research group [79] reported that glutamate-liposomes can be targeted to LAT1, lysine-liposomes can be targeted to ATB^0,+^, and, inspiringly, tyrosine-liposomes can be simultaneously targeted to LAT1 and ATB^0,+^. Based on these results, the authors further investigated tyrosine-liposomes in a different study [80], using them as carriers for irinotecan. The nanosystem displayed high encapsulation efficiency, a stable drug release profile, excellent tumor site accumulation, strong antitumor activity, and attenuated side effects compared to ligand-exposure liposomes.

Interesting results have also been obtained by encapsulating traditional oriental medicine into liposomes. In this respect, Zhang et al. [81] have developed and evaluated bufalin-loaded RGD targeted PEGylated liposome (L-RGD-PEG-BF). The as-functionalized particles were noticed to release therapeutic cargo in a slower manner than conventional liposomes, ensuring a prolonged and sustained release. Moreover, the authors reported high inhibition on A549 cells proliferation and much lower IC_50_ than for pure bufalin, L-RGD-PEG-BF showing an active targeting property on A549 cells. Thus, this nanosystem holds promise for translation in Chinese medicine.

In contrast, Anilkumar et al. [82] developed multifunctional liposomes for dual-targeted (magnetic and ligand) and dual-mode (photothermal/photodynamic) cancer therapy. This was achieved by encapsulating indocyanine green into magnetic liposomes coated with citric acid and hyaluronic acid–PEG. The as-designed liposome-based formulation exhibited a highly efficient photothermal effect, enhanced cytotoxicity against human glioblastoma cells after 4 min exposure to NIR laser, and good accumulation at tumor sites.

One more innovative strategy was proposed by Liu et al. [83]. The authors encapsulated doxorubicin into Erbitux-conjugated, thermal-sensitive, multifunctional liposomes based on manganese-doped, magnetism-engineered iron oxide nanoparticles and gold nanorods. This interesting combination of elements allowed for efficient photothermal therapy and magnetic resonance (MR) imaging. The EGFR-targeting nanosystem was specifically bound to A431 tumor cells and promoted tumor destruction by laser activation without generating significant morphological damage to normal tissues. Therefore, this system has potential as a diagnostic and therapeutic platform for EGFR-overexpressing tumors.

Another example of tumor-targeted liposomes was proposed by He and colleagues [84]. The researchers synthesized a targeted binary-drug liposome modified with lactoferrin, whose activity was enhanced by the in situ formation of albumin corona. Thus, they achieved a dual-targeting effect on the receptors of both LRP-1 and SPARC that were overexpressed in tumor cells and immune cells. The newly designed constructs effectively suppressed the crosstalk between tumor metabolism and immune evasion by glycolysis inhibition and immune normalization, offering a promising approach for remodeling TME.

Alternatively, Yu et al. [85] constructed a complex nanoplatform responsive to the membrane biomarker FAP-α on CAFs and NIR laser irradiation. The researchers loaded a small-sized albumin nanoparticle of paclitaxel and a photothermal agent (i.e., IR-780) into CAP-modified thermosensitive liposomes. The as-designed nanosystem increased drug retention in solid tumors and promoted medicine release in deep tumor regions, enhancing its anticancer activity. Therefore, these platforms could effectively serve as a treatment alternative for pancreatic ductal carcinoma.

### 3.4. Cubosomes

Cubosomes are another class of nanocarriers with promising theranostic efficiency [86]. These materials are self-assembled, honeycomb-like, three-dimensional structures in the bicontinuous cubic liquid crystalline phase that provide advantageous properties for developing advanced delivery systems. These beneficial features include a large interfacial area; a relatively simple preparation method; an ability to encapsulate hydrophobic, hydrophilic, and amphiphilic moieties; biodegradability; and a targeted and controlled release of bioactive freight. Thus, it is no surprise that cubosomes have started to receive an increasing amount of scientific attention for fabricating tumor-targeted vehicles with different internal cubic structures, compositions, and drug-loading modalities [6,47,87].

For instance, Patil et al. [88] fabricated inhalable, bedaquiline-loaded cubosomes as nanomedicines against non-small cell lung cancer. In vitro, the cubosomal formulation displayed an initial burst release followed by a sustained release pattern for 72 h. However, in vivo, cubosomes may undergo enzymatic degradation and endocytosis, which contribute to a faster release of the carried drug. These nanostructures were noted to suppress cell proliferation and to inhibit colony formation and cancer metastasis in vitro, having enhanced anticancer activity compared to free drugs. Moreover, this formulation exhibited an optimal aerodynamic diameter, excellent deep lung deposition after nebulization, and rapid cell internalization.

An interesting approach was proposed by Faria et al. [89], who have encapsulated elesclomol (ELC) into monoolein-based cubosomes stabilized with Pluronic F127. The nanostructures accumulated in proximity to the mitochondria with a sub-micrometer distance, inducing cytotoxicity through ROS. The researchers also investigated the activity of cubosomes loaded with pre-complexed copper-ELC, reporting improved cytotoxicity and promising features for systemic administration.

Alternatively, Bazylińska et al. [90] stabilized monoolein-based cubosomes with phospholipids and used propylene glycol as a hydrotrope. These nanocarriers were employed for the delivery of photosensitizers (i.e., chlorin e6 and meso-tetraphenylporphine-Mn(III) chloride), demonstrating effective internalization into melanoma cell lines, considerable cytotoxicity under photoirradiation, and very low toxicity in the “dark” condition.

More recently, Saber and colleagues [91] loaded cubosomes with albendazole with the goal of modulating ERK1/2-HIF-1α-p300/CREB interactions. The nanocarriers improved the bioavailability of the drug, leading to antiangiogenic and antimetastatic activity. The authors concluded that disrupting this interplay generates a new therapeutic target for managing hepatocellular carcinoma.

### 3.5. Lipid Nanoparticles

Lipid nanoparticles, in their various forms (e.g., solid-lipid nanoparticles, lipid-drug complexes, nanostructured lipid carriers, polymer-lipid conjugates), present appealing properties for creating tumor-targeting nanosystems with different applications [92]. The advantages of lipid nanoparticles include biocompatibility, biodegradability, bioavailable colloidal carriers, preparation through simple and safe techniques, desirable drug encapsulation, sustained and controlled cargo release, and the possibility of active targeting [6,93].

Taking into account these useful properties, Wang and colleagues [94] developed paclitaxel- and naringenin-loaded solid-lipid nanoparticles (SLNs) as an innovative treatment for glioblastoma multiforme. In addition, the authors functionalized the surface of SLNs with a cyclic RGD peptide sequence (Arg-Gly-Asp). The matrix structure of the nanocarrier was noted to provide a sustained drug release behavior, while its nano-size and lipohilic characteristics facilitated nanosystem uptake via intercellular and paracellular pathways. Hence, the as-designed particles displayed higher toxicity than free drugs, superior cellular uptake, enhanced oral bioavailability, and a better chemoprotective effect. Similarly, Arduino et al. [95] functionalized SLNs with a tumor-homing peptide (i.e., iRGD) for the targeted release of paclitaxel. This surface modification also allowed for more efficient tumor targeting and penetration, better cellular uptake, and enhanced anticancer activity.

Another study conducted by Jang et al. [96] demonstrated that SLNs are proper carriers for camptothecin (CPT) as well. The nanoparticles were stabilized with pegylated phospholipids and formulated for intravenous administration. These nanomedicines showed remarkable tumor targeting, prolonged blood circulation, and tumor growth inhibition. Moreover, the researchers reported that the pre-injection of bare SLNs before drug-loaded particles reduced the accumulation of CPT-SLNs in reticuloendothelial system-rich tissues and organs, enhancing tumor targeting, improving pharmacokinetic parameters, and increasing the antitumor performance of CPT-encapsulated delivery systems.

Alternatively, Wang et al. [97] proposed the use of ApopB-100-based, targeted, low-density, lipoprotein (LDL)-based nanoparticles for the codelivery of sorafenib and dihydroartemisinin. The LDL-based drug delivery carrier could target LDLR-overexpressed cells, enhancing the intracellular level of drugs in specific tumor cells. The authors obtained a synergistic anticancer effect in liver cancer cells, noting a significant decrease in cell viability compared to either single drug. Moreover, a robust antitumor response and delayed tumor growth were reported, recommending these nanoplatforms as specific tumor-targeting treatments.

In contrast, Rosenblum and colleagues [98] utilized lipid nanoparticles for the delivery of Cas9 mRNA and sgRNAs. By using a single, intracerebral injection of CRISPR-LNPs against PLK1 on aggressive orthotopic glioblastoma, up to ~70% of gene editing was registered in vivo, leading to tumor growth inhibition and survival improvement. In addition, the researchers also created EGFR-targeted particles for reaching disseminated tumors, causing their selective uptake into disseminated ovarian tumors and leading to promising results when evaluating their potential in vivo.

For an even better loading of hydrophilic drugs, lipid-polymer hybrid nanoparticles (LPNs) appear to be a highly convenient solution [2]. For instance, Chen et al. [99] combined the advantages of a polymeric core and a lipid shell for the codelivery of curcumin and cabazitaxel. The authors employed aptamer-functionalization to endow particles with a targeting ability with regard to prostate cancer cells. Thus, the nanosystems presented a good cell inhibition ability, high tumor accumulation, and remarkable tumor inhibition efficiency, and are a potential strategy against prostate cancer.

Alternatively, Wang and colleagues [100] synthesized and tested RGD-modified LPNs for the codelivery of redox-sensitive paclitaxel prodrug and cisplatin. Using RGD peptide as a targeting ligand offered the nanoplatform a high affinity for α_v_β_3_ integrin overexpressing lung tumor cells. Compared to free drugs, these nanoparticles exhibited significantly higher antitumor activity and lower systemic toxicity, and are promising candidates for lung cancer therapy.

### 3.6. Polymeric Nanoparticles

The variety and versatility of polymers have drawn increasing research interest towards fabricating performant nanocarriers for a broad range of cargos. Polymeric-based nanoparticles can encapsulate, protect, and deliver different loads, counting active pharmaceutical ingredients, nucleic acids, imaging moieties, and other biomolecules, offering advantageous properties for realizing tumor-targeting vehicles [101]. Through varied modalities of surface functionalization, polymeric nanoparticles can decrease drug clearance, enhance load stability and solubility, and prolong the half-life of the carried agents, thus allowing optimal target site accumulation [102].

**Figure 8:**
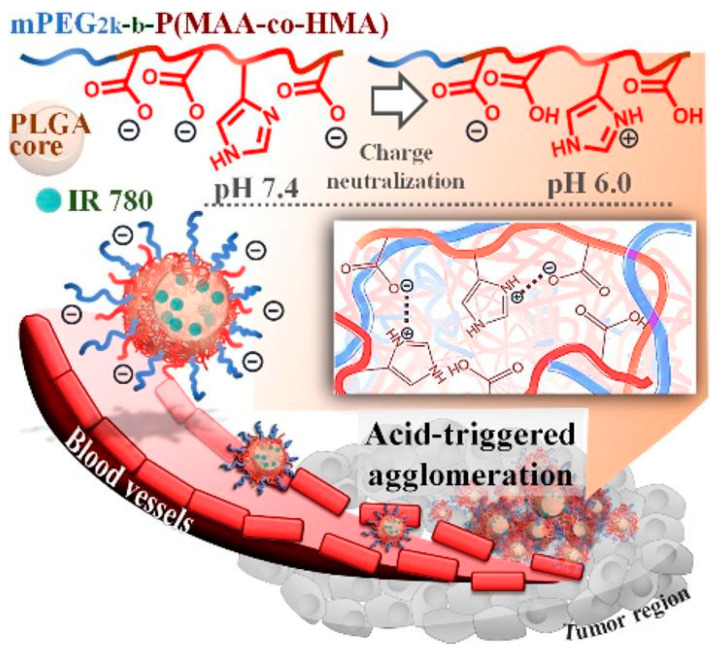
Schematic representation of the enhanced tumor retention of nanovehicles designed by Lu et al. by virtue of acid-triggered surface charge neutralization and agglomeration. Reprinted with permission from [103]. Copyright 2020, Elsevier.

For instance, Lu et al. [103] created polymeric vehicles for the transportation of an IR780 photothermal agent. The authors used PLGA cores coated with zwitterionic diblock copolymers (i.e., methoxypoly(ethylene glycol)-b-poly(methacrylic acid-co-histamine methacryamide), mPEG-b-P(MAA-co-HMA)) to create pH-responsive nanocarriers. The as-designed system took advantage of the weak acidity of TME, leading to enhanced cellular uptake by TRAMP-C1 cells and improved tumor retention capabilities (Figure 8).

More recently, Wang et al. [104] also tackled the potential of pH-responsive polymeric systems. The researchers fabricated nanocarriers made of Mal-PAH-PEG-DMMA/poly (ethylene imine)—poly(ε-caprolactone) block polymers and loaded them with docetaxel and IR825 photosensitizer. These complex delivery systems enhanced cellular uptake, increased drug release in response to acidic conditions and NIR irradiation, and provided excellent photothermal conversion efficiency. This combined, chemo-photothermal therapy displayed more efficient tumor ablation than either therapy alone, while also maintaining good biocompatibility and safety.

Alternatively, Li and colleagues [105] developed L-phenylalanine-based poly(ester amide) nanoparticles loaded with NSC23766. These vehicles released their cargo in a pH-responsive manner, leading to a fast uptake by PC3 cells and significant inhibition of PCa cell proliferation. In vivo studies further proved that the administration of these particles intravenously results in increased drug concentration and prolonged retention at the tumor site, being a suitable candidate for treating prostate cancer.

An interesting study conducted by Zhang et al. [106] proposed the use of polymeric nanoparticles loaded with an immunomodulator (i.e., R848) that can spontaneously target the mitochondria of tumor cells. The particles were functionalized with the tumor-targeting, cyclic Arg-Gly-Asp (cRGD) peptide and labeled with Changsha Red. These modifications allowed for the induction of hyperthermia specifically in tumor tissues, efficiently damaging mitochondria in cancer cells and promoting the release of tumor-associated antigens. Moreover, combined with the immune checkpoint blockade, the activated antitumor immune response can effectively suppress distant tumors and overcome tumor recurrence and metastasis, leading to long-term antitumor outcomes.

In contrast, Liu et al. [107] developed anticancer delivery systems based on molecularly imprinted polymers. The authors used dopamine as a functional monomer and photothermal agent, doxorubicin as a chemotherapeutic agent, zeoliticimidazolate framework-8 as a drug carrier, and the epitope of EGFR as template molecules. These complex nanoparticles have the ability to recognize EGFR-overexpressing cancer cells and produce synergistic chemo-photothermal effects under NIR irradiation. Altogether, the selective targeting, intelligent drug release capacity, biocompatibility, and minimal toxicity to normal cells recommend these nanosystems as multifunctional platforms for cancer treatment.

One more recent innovative drug delivery strategy was proposed by Yakati et al. [108]. The researchers employed the encapsulation of paclitaxel into PLGA nanoparticles functionalized with a tumor-homing peptide (i.e., CPKSNNGVC, CPK in short). These polymeric nanocarriers can target both monocarboxylate transporter 1 (MCT1) receptor-positive cancer cells and angiogenic endothelial cells, demonstrating preferential cellular uptake and the apoptosis-mediated cell death of MCT1 receptor-overexpressing colorectal cancer cells, while also inhibiting the formation of new blood vessels. The described effects also contribute the sustained release of the cargo, whose profile shows an initial burst, followed by a steady release over the course of analysis (i.e., 8 days).

A distinct type of polymer that is increasingly being investigated for developing targeted drug and gene delivery systems is represented by dendrimers. In comparison with conventional polymers, dendrimers present a well-defined 3D structure whose spherical shape, surface functionality, and size can be precisely tailored to specific applications. Thus, these special nanomaterials allow the encapsulation of desired compounds inside their cavities or covalent conjugation with a cleavable linker for TME-targeting [109,110,111,112].

**Figure 9:**
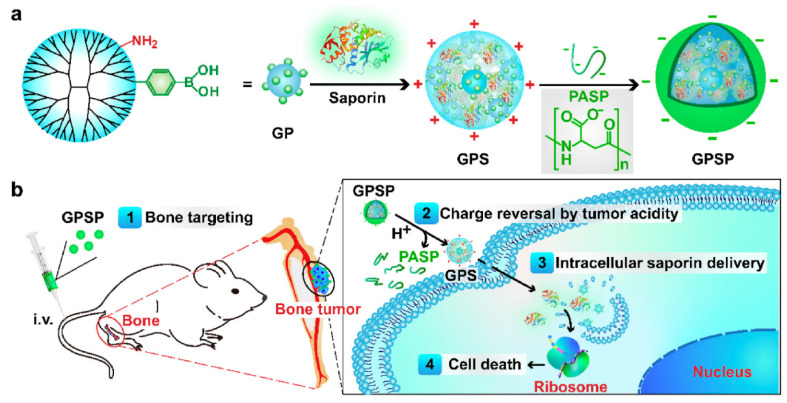
(**a**) Design of bone-targeted, protein-functionalized, dendrimer-based nanomedicine for treating malignant bone tumors. (**b**) Schematic representation of the mode of action of the developed nanosystem. Reprinted from an open-access source [113]. Abbreviations: GP—G5-phenylboronic acid; GPS—GP-saporin complex; GPSP—GPS-PASP ternary complex; i.v.—intravenously; PASP—poly-(α, β)-DL-aspartic acid.

For example, Yan and colleagues [113] recently developed a dendrimer-based, bone-targeted protein nanomedicine for the treatment of malignant bone tumors (Figure 9). The ternary complex nanoparticles they prepared displayed high bone accumulation, protein (i.e., saporin) release triggered by tumor extracellular acidity, and intracellular delivery, which led to ribosome inactivation in cancer cells. Thus, this approach may represent a viable strategy in treating osteosarcoma and bone metastasis.

A different study investigated the potential of dendrimer-based nanomedicines in treating glioblastoma. Sharma and colleagues [114] prepared sugar (i.e., glucose, mannose, or galactose)-conjugated, hydroxyl-terminated, polyamide-amine dendrimers to target upregulated sugar transporters. Different behaviors were noted for the different sugars used. Glucose-modified particles enhanced the targeting of TAMs and microglia by improving brain penetration and cellular internalization; galactose-modified dendrimers instead targeted galectins on glioblastoma tumor cells; and mannose functionalization altered the kinetics of dendrimer accumulation within the tumor.

Pursuing a similar purpose, Liaw et al. [110] prepared dendrimer-triptolide conjugates that improve phenotype switching in TAMs from pro- towards antitumor expression in glioblastoma. The system was reported to considerably ameliorate tumor burden compared to free triptolide, while also diminishing the hepatic and cardiac toxicities associated with this substance. Thus, this delivery strategy holds promise for attenuating the systemic toxicities of chemotherapeutics without compromising their anticancer activity.

Researchers have also used dendrimers as multifunctional doxorubicin carriers. In this respect, Zhang et al. [115] loaded this chemotherapeutic drug into polyamide–amine dendrimer-grafted, persistent luminescence nanoparticles functionalized with aptamer AS1411. These nanosystems could specifically bind to the overexpressed nucleolin on the membrane of tumor cells, enhancing intracellular accumulation at the desired site, inducing apoptosis in HeLa cancer cells, and inhibiting tumor growth.

Alternatively, Xiong et al. [116] developed a theranostic, dendrimer-based lipid nanoplatform containing PEGylated BODIPY dyes (PBD) for mRNA delivery and NIR imaging. The system successfully mediated mRNA expression in tumors while simultaneously illuminating these tissues through pH-responsive NIR imaging. Therefore, the designed platform foresees potential in the combined detection and treatment of cancer.

### 3.7. Micelles

**Figure 10:**
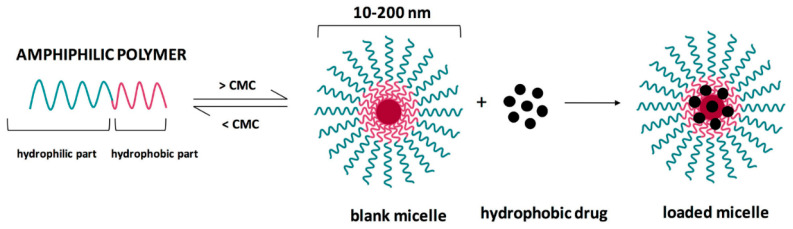
Schematic representation of polymeric micelles. Reprinted from an open-access source [118]. Abbreviation: CMC—critical micellar concentration.

Micelles represent yet another category of nanomaterials for developing efficient tumor-targeting modalities for cancer management. These nanoconstructs are characterized by a core-shell structure created through the self-assembly of amphiphilic block copolymers in an aqueous solution that can improve the solubility of carried moieties, potentiate the therapeutic efficacy of the drugs, and diminish their adverse effects on healthy tissues (Figure 10) [117]. Advantageously for cancer therapy, many smart micelles that are responsive to internal or external stimuli have been created for drug delivery to tumor tissues [118,119].

Particular attention has been drawn to creating doxorubicin micelle-based drug delivery vehicles. In this respect, Guo et al. [120] entrapped this chemotherapeutic agent in daptomycin micelles, creating a pH-responsive nanosystem. Studies revealed good stability in fibrinogen solution, controlled release in acidic media, enhanced cytotoxicity compared to free drugs, an excellent tumor inhibition effect, and good in vivo biocompatibility. Alternatively, Tian and colleagues [121] encapsulated doxorubicin in aptamer-modified polymeric micelles targeting pancreatic cancer cells. The nanostructures successfully released the drug, exhibiting better tumor penetration than their counterparts without aptamer modification. Another approach was proposed by Wan et al. [122], who created a prodrug by covalently linking D-α-tocopherol polyethylene glycol succinate (TPGS3350), peptide, and doxorubicin. This prodrug further self-assembled into micelles, physically encapsulating the anticancer agent. At the tumor site, the nanostructure disassembled, exposing the targeting molecule folate and entering the cell via endocytosis. The nanosystem exhibited excellent antitumor efficiency and low side effects in healthy tissues, representing a promising strategy for cancer therapy.

A different method was proposed by Domiński et al. [123]. The authors prepared micelles from an amphiphilic triblock copolymer (i.e., poly(ethylene glycol)-b-polycarbonate-b-oligo([R]-3-hydroxybutyrate) for the delivery of doxorubicin and 8-hydroxyquinoline glucose- and galactose-conjugates. These nanosystems preferentially released the drug cargo at an acidic pH and significantly inhibited the proliferation of MCF-7 and HCT-116 cells, revealing new possibilities for developing more efficient cancer therapies.

Other drugs have also been considered for micelle encapsulation. For instance, Sethi et al. [124] used carbamoylethyl pullulan-grafted palmitic acid self-assembled micelles for the delivery of raloxifene to mammary carcinoma. The authors reported a pH-dependent drug release, enhanced drug concentration in the targeted tumor, and diminished concentration in other tissues, as compared to the free chemotherapeutic. Alternatively, Andrade et al. [125] loaded niclosamide into CD44v6-targeted polymeric micelles in an effort to create a better treatment approach for colorectal cancer. The researchers obtained encouraging results, as the nanosystems accumulated in tumor tissues and considerably reduced circulating tumor cells in vivo. Furthermore, Zhang and colleagues [126] created tumor-targeting micelles for the delivery of paclitaxel. The targeting ability was endowed via modification with folic acid and α-tocopherol succinate-conjugated hyaluronic acid. These nanoconstructs improved delivery accuracy to the target site, enhanced antitumor activity, and reduced toxicity in healthy tissues. Other interesting studies have also reported the successful preparation of various micelle-based delivery systems encapsulated with telmisartan [127], camptothecin [128], gemcitabine, and deoxycholic acid [129], etoposide, and all-trans retinoic acid [130], that could serve as performant, tumor-targeting nanoplatforms.

Micelles can also be employed in developing performant nanomaterials for phototherapy. Deng et al. [131] reported the formation of micelles from an amphiphilic iridium-based photosensitizer (i.e., C14-IP2000) loaded with a photothermal drug (i.e., zinc(II)-2,9,16,23-(tetra-t-butyl)phthalocyanine). These nanoconstructs exhibited effective blood circulation, passive tumor targeting capacity, exceptional photodynamic conversion abilities, and significant photothermal conversion capability, rendering them suitable for combined tumor ablation.

### 3.8. Virus-Like and Virus-Based Nanomaterials

Virus-like particles (VLPs) are multimeric nanostructures that have attracted interest for targeted-delivery strategies as well. They consist of self-assembled, non-replicative, and non-infectious protein particles that mimic original, wild-type viruses without (or with only a fragment of) the viral genome. Thus, these nanoparticles are safe alternatives for creating drug and vaccine carriers, lacking the risk of replication, recombining, or reverting to virulent stages [132].

**Figure 11:**
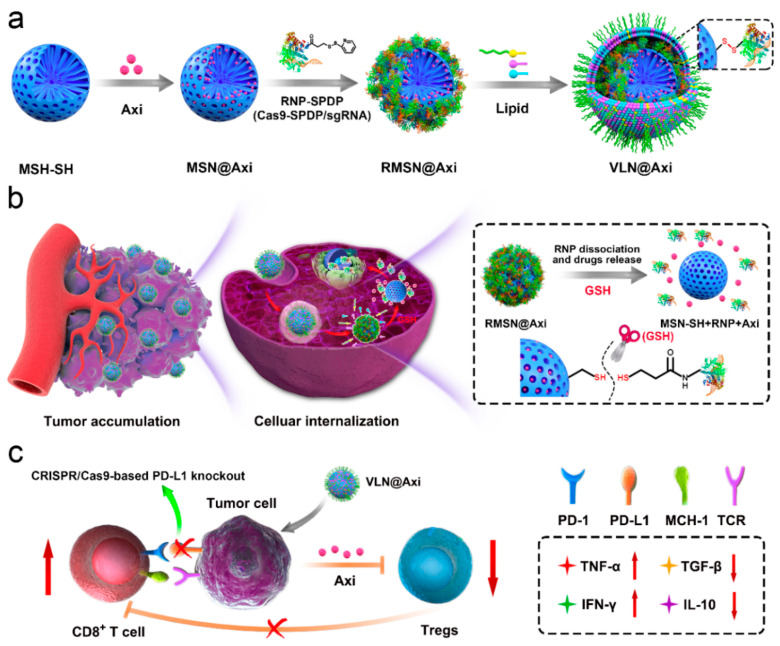
Schematic representation of the (**a**) synthesis, (**b**) delivery process, and (**c**) mode of action of VLPs designed by Liu et al. Reprinted with permission from [133]. Copyright 2020, Elsevier. Abbreviations: Axi—axitinib; GSH—glutathione; MSN—mesoporous silicon nanoparticle; RMSN—ribonucleoprotein-conjugated MSN; VLN—virus-like nanoparticle.

In this respect, Liu et al. [133] coloaded small molecule drugs and a CRISPR/Cas9 system into a mesoporous silica nanoparticle (MSN)-based core coated with a lipid shell. The freight was released in response to the reductive TME, leading to the concerted regulation of multiple cancer-associated pathways and the suppression of melanoma growth in vivo (Figure 11). As virus-like nanoparticles can co-deliver almost any combination of sgRNAs and small molecule drugs to tumors, the developed approach could be a universal platform for creating synergistic therapies against various malignant tumors.

Campbell et al. [134] fabricated VLPs to recombinantly express murine and conjugated them with an aberrantly glycosylated mucin-1 (MUC1) peptide survivin. The authors reported that the co-delivery of two tumor antigens on VLPs leads to enhanced survival compared to VLPs delivering either antigen alone. Nonetheless, supplementary research is required in regard to breaking tolerance when targeted tumor antigens are expressed as endogenous self-proteins.

A promising VLP-based strategy was proposed by Simons et al. [135]. The authors synthesized a VLP vaccine made of bovine papillomavirus L1 protein, engineered to display surface docking sites and decorated with peptides encoding T cell epitopes from two prostate cancer-associated tumor antigens and a neo-antigen stimulator of prostatic adenocarcinoma-specific T cells. This treatment significantly reduced tumor burden and increased CD3 + and CD8 + T cell infiltration into tumor tissue of advanced prostate cancer animal models.

Plant viruses have also recently been viewed as a safe and promising alternative for drug delivery [136,137]. Moreover, plant virus nanoparticles exhibit intrinsic immune-stimulatory abilities, and are being researched as immune adjuvants with regard to enhancing antitumor immune response [138].

In this respect, Alemzadeh et al. [136] developed a nanocarrier for doxorubicin made of Johnson grass chlorotic stripe mosaic virus (JgCSMV) conjugated to folic acid. This complex displayed a sustained drug release at the target site and improved doxorubicin uptake in cancer cells, leading to tumor growth inhibition and reduced cardiotoxicity of athymic mice bearing human breast cancer xenografts.

In contrast, Franke et al. [139] prepared tobacco mosaic virus (TMV) conjugated with cisplatin as a nanomedicine against platinum-resistant ovarian cancer. The nanostructures were efficiently taken up by cancer cells, resulting in superior cytotoxicity and DNA double-strand breakage in platinum-sensitive and platinum-resistant cancer cells compared to the free drug. Nonetheless, the nanosystem could benefit from additional modifications to its surface functional groups to improve its targeting ability and/or impart new functionality.

In another study, Gamper et al. [140] created a functionalized nanoscaffold by using the coat protein of TMV as a carrier for a highly hydrophobic peptide that targets the transmembrane domain of the Neuropilin-1 (NRP1) receptor in cancer cells. The designed platform was noted to bind to NRP1 in cancer cells, disrupt its complex formation with PlexA1, downstream Akt survival signaling, and inhibit angiogenesis and cell migration.

Alternatively, Shukla et al. [141] fabricated monomethyl auristatin delivery vehicles for Non-Hodgkin’s B cell lymphomas based on potato virus X (PVX). PVX was noted to have a binding affinity towards malignant B cells, allowing the platform to target desired tissues and effectively deliver the drug cargo. Moreover, the nanosystem inhibited lymphoma growth and improved the survival of mouse models, and is considered a promising drug delivery platform for B cell malignancies.

### 3.9. Exosomes

**Figure 12:**
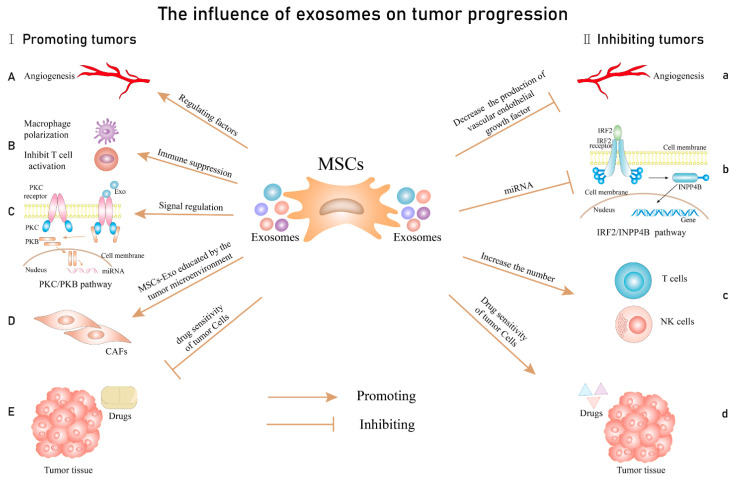
The influence of exosomes on tumor progression. Promoting tumors: (**A**) including the regulation of the secretion of mediators of angiogenesis; (**B**) promoting the immune escape by regulating macrophage polarization and inhibiting T cell activation; (**C**) stimulates tumor cell proliferation by affecting signaling pathways; (**D**) the tumor microenvironment mediates cancer-associated fibroblast (CAF) formation by educating MSCs-Exo; (**E**): MSCs-Exo increase drug resistance. Inhibiting tumors: (**a**) inhibition of angiogenesis; (**b**) inhibition of tumor proliferation through miRNA-mediated signaling pathways; (**c**) increase the number and sensitivity of T cells and NK cells; (**d**) improving drug sensitivity. Reprinted from an open-access source [145].

Recent studies have started to investigate exosomes as viable nanomaterials for cancer diagnosis and therapy. These nanostructures are endogenous particles secreted by various cells and absorbed by recipient cells, whose unique structural and compositional features endow them with low cytotoxicity and the ability to overcome biological barriers and escape immune surveillance. Exosomes can stabilize encapsulated nucleic acid, proteins, or other therapeutic agents; penetrate cell membranes; and release their cargo to the desired site [142]. Moreover, the exosomal membrane presents numerous protein molecules, nucleic acids, proinflammatory factors, cytokines, and transcription factor receptors on its surface, allowing the involvement of exosomes in many cellular activities [143,144,145] (Figure 12).

Taking into account the great potential of these nanostructures, Wang and colleagues [143] proposed the combined use of designer exosomes and chemo/gene/photothermal therapy. The researchers loaded exosomes with doxorubicin and coated them with magnetic nanoparticles conjugated with molecular beacons that can target miR-21 for responsive molecular imaging. The nanocarriers can be guided to the tumor site via the application of an external magnetic field, and while under NIR irradiation, they induce localized hyperthermia and release the drug. This combined therapy was reported to dramatically reduce tumor size (97.57%), and is a promising strategy for developing next-generation precision cancer nanomedicines.

In a similar manner, Kwon et al. [144] developed a novel nanostructure for the treatment of colorectal cancer. The scientists used exosomes isolated from the tumor cell line as a doxorubicin carrier, functionalized them with folic acid, and attached magnetic nanoparticles to their surface. The nanosystem demonstrated increased apoptosis and excellent tumor growth inhibition ability, and is a potential candidate for effective cancer therapy. Alternatively, Pei and colleagues [146] proposed a different approach against colorectal cancer. The authors reported a combination therapeutic strategy that dually inhibits FGL1 and TGF-β1 towards simultaneously blocking the immune checkpoint and modulating TME. In this respect, they have developed a cRGD-modified exosome with high siFGL1 and siTGF-β1 loading efficiency, which increased the number of tumor infiltration CD8+ T cells while decreasing the number of immunosuppressive cells.

Alternatively, Zhou et al. [147] prepared an exosome-based delivery system for augmenting pancreatic ductal adenocarcinoma immunotherapy and reversing the tumor immunosuppression of M2-like TAMs upon disruption of galectin-9/dectin 1 axis. The platform was created from bone marrow mesenchymal stem cell exosomes, loaded with galectin-9 siRNA, and surface-modified with oxaliplatin prodrug. The nanosystem presented excellent tumor targeting efficacy and elicited antitumor effects by tumor-suppressive macrophage polarization, cytotoxic T lymphocytes recruitment, and Treg downregulation.

More recently, Huang and colleagues [148] combined exosome technology with lncRNA MEG3 for the tumor-targeting therapy of osteosarcoma. Specifically, the authors loaded MEG3 into exosomes modified with c(RGDyK) peptide that could more effectively deliver to the target bone cancer cells, both in vitro and in vivo. Thus, these systems have promising therapeutic effects for osteosarcoma.

**Figure 13:**
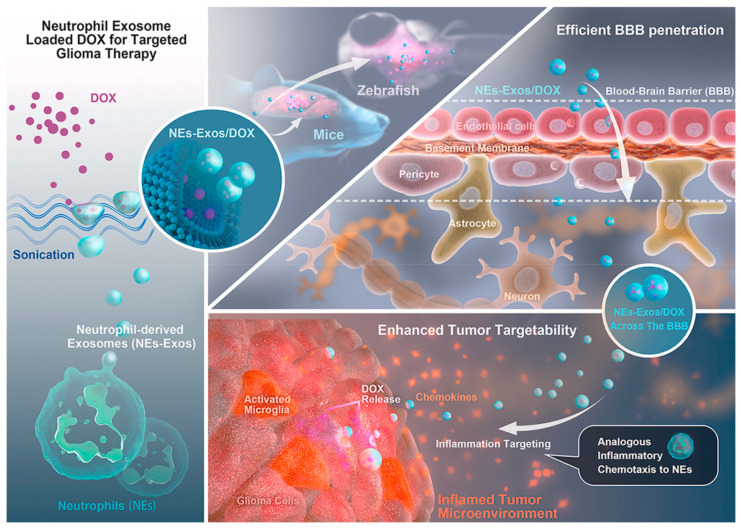
Schematic representation of doxorubicin loading into neutrophil-exosomes, BBB crossing of the nanosystem, and inflammatory stimuli-responsive drug delivery. Reprinted with permission from [149]. Copyright 2021, Elsevier. Abbreviations: BBB—blood-brain barrier; DOX—doxorubicin.

Another research group [149] created an innovative nanocarrier system for the delivery of doxorubicin to treat glioma. The authors designed bioinspired neutrophil-exosomes with inherent inflammatory chemotaxis and excellent BBB penetration abilities (Figure 13). Thus, this nanoplatform shows significant potential for the clinical treatment of glioma and other solid tumor or brain diseases.

A different study, conducted by Zhu et al. [150], reported a novel radiosensitizer consisting of manganese carbonyl-loaded exosome nanovesicles. The nanoparticles allowed for controlled robust carbon monoxide evolution and subsequential ROS generation under X-ray irradiation, facilitating tumor growth inhibition under very low-dose radiotherapy.

Alternatively, Molavipordanjani et al. [151] created ^99m^Tc-radiolabel HER2-targeted exosomes for tumor imaging. The nanosystems exhibited higher affinity towards SKOV-3 cells than to MCF-7, HT29, U87-MG, or A549 cell lines, demonstrating preferential targeting towards ovarian adenocarcinoma. This allowed for accumulation at the desired site and for visualization of the tumor in SKOV-3 tumor-bearing nude mouse models.

### 3.10. Cell Membrane-Coated Nanomaterials

A recently emerged strategy uses cell membrane coatings to create biomimetic nanoparticles with functions and properties inherent to source cells for various biomedical applications [152]. In this context, particular attention has been drawn to preparing drug-delivery nanoplatforms covered with various membranes from blood cells, tumor-specialized cells, bacterial cells, or hybrid engineered membranes [153].

For instance, Li et al. [154] proposed a new type of antineoplastic agent. The authors used a two-dimensional graphene oxide, loaded with indocyanine green and doxorubicin, and covered with a red blood cell (RBC) membrane, functionalized with folic acid. The endogenous nature of the shell endows the delivery system with the ability to evade clearance by the reticuloendothelial system, while the targeting ligand allows selective recognition of tumor cells via a lipid-insertion approach. Thus, this strategy could serve as a promising tumor-targeted chemo-photothermal agent in the clinic.

Wen et al. [155] also tackled the benefits of RBC membranes. The researchers functionalized them with cRGD and used them as coatings for gefitinib-loaded albumin nanoparticles, creating a biomimetic delivery system. The as-designed nanoplatform inhibited the growth of A549 cells in vitro in a dose- and time-dependent manner, decreased tumor weight and volume, and prolonged survival time. Moreover, through ^99^Tc labeling, the nanosystem allowed for real-time tumor imaging.

In addition, important results have been reported when fusing together RBC membranes with the membranes of cancer cells. In this respect, Jiang et al. [152] created a hybrid RBC-MCF-7 coating and used it to camouflage melanin nanoparticles. The delivery system demonstrated prolonged blood circulation, excellent tumor accumulation, and effective phototherapy effects. Following a similar strategy, Xiong et al. [156] fused a murine-derived ID8 ovarian cancer cell membrane with an RBC membrane to camouflage indocyanine green-loaded magnetic nanoparticles. The particles displayed a highly specific self-recognition of ID8 cells and prolonged circulation lifetime. Moreover, the nanosystem was noted to activate specific immunity and photothermally induce tumor necrosis, and is a promising candidate for synergistic photothermal immunotherapy against ovarian cancer.

Besides RBCs, other membranes could be utilized as well. Wu et al. [157] and Zhou et al. [158] employed platelet membranes for coating doxorubicin-loaded polypyrrole nanoparticles (PLT-PPy–DOX) and disulfide-containing biodegradable PLGA conjugate nanoparticles (rVAR2-PM/PLGA-ss-HA). Under laser irradiation, the PLT-PPy–DOX nanoplatforms released the drug cargo and generated hyperthermia in tumor tissues, suppressing primary tumor growth and inhibiting tumor metastases in hepatocellular carcinoma. In contrast, rVAR2-PM/PLGA-ss-HA released the chemotherapeutic drugs in response to the high intracellular concentration of reduced glutathione present in the TME, and is a promising strategy for treating various primary and metastatic cancer types.

Alternatively, Wang et al. [159] prepared leukocyte membrane-coated gallium nanoswimmers that can be propelled via ultrasound. The nanoplatform presented anti-biofouling, cancer cell recognition, and targeting properties; integrating imaging; drug delivery; and photothermal cancer treatment capacities.

Cancer cell membranes also offer advantageous properties for creating tumor-targeting nanoparticles. For example, Ren et al. [160] employed mouse colon cancer CT26 cell membranes to enhance the biocompatibility of bismuth nanoparticles and endow them with a targeting ability. These particles could effectively ablate colon cancer cells via photothermal therapy, as the tumors tended to be eradicated after 12 days. Similarly, Yao et al. [161] used CT26 cells membranes as a delivery vehicle for cyclopeptide RA-V (to directly kill tumor cells) and PD-1/PD-L1 blockade inhibitor (to produce antitumor immune responses). Their system ingeniously and effectively combined the advantages of chemotherapy and checkpoint blockade-based immunotherapy, leading to promising therapeutic efficacy against hypoxic tumor cells.

**Figure 14:**
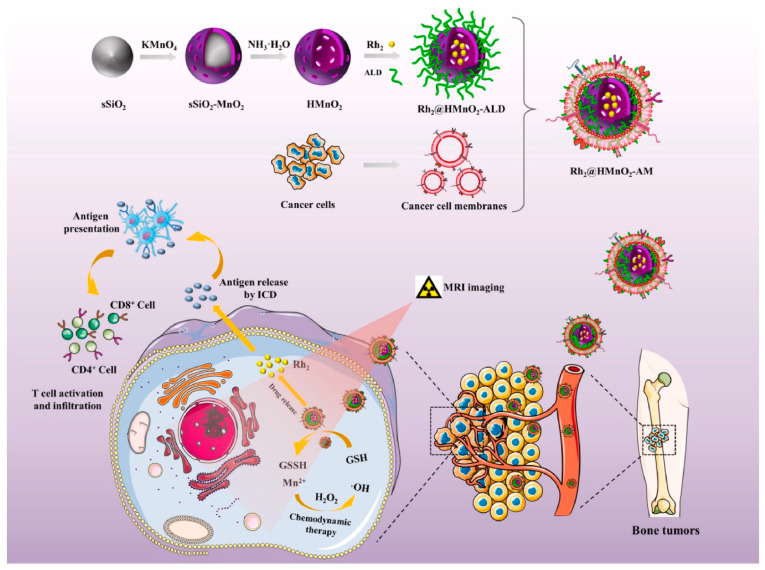
Rh2@HMnO_2_-AM synthesis procedure and the mechanism of MRI-guided immuno-chemodynamic synergistic osteosarcoma therapy. Reprinted from an open-access source [162].

Alternatively, Fu and colleagues [162] developed ALD/K7M2 cell membrane-coated hollow manganese dioxide (HMnO_2_) nanoparticles for the delivery of Ginsenoside Rh2 (Figure 14). The as-designed nanoplatforms are suitable for MRI-guided immuno-chemodynamic combination therapy against osteosarcoma.

Another study conducted by Gong et al. [153] reported the use of a hybrid membrane fabricated from the components of RAW264.7 and 4T1 cells membranes for coating doxorubicin-encapsulated PLGA nanoparticles. The as-designed nanoparticles were employed in treating lung metastases from breast cancer, exhibiting excellent targeting ability and chemotherapeutic potential.

Wang et al. [163] tackled yet another different approach. The authors prepared a hybrid membrane by combining a bacterial membrane vesicle and B16-F10 cancer cell membrane as a coating material for hollow polydopamine nanoparticles. The system benefited from the synergy of the therapeutic potential of immunotherapy and photothermal therapy, leading to enhanced tumor efficiency against melanoma. Moreover, the system could also provide adaptability to imaging applications if application-specific functions are incorporated.

## 4. Conclusions

Cancer represents an ongoing challenge for the scientific community, posing a tremendous burden on patients and healthcare systems worldwide. Several conventional and adjuvant anticancer therapies are currently used in clinical practice with different degrees of success. Unfortunately, however, most of these treatment strategies may result in severe adverse effects or/and unsatisfactory therapeutic outcomes.

Attempting to overcome these limitations, nanomedicine has started to gain increasing popularity in cancer management. Particular interest and promising results have been registered in developing a broad range of tumor-targeting nanoparticles for cancer therapeutic applications.

A variety of materials have been investigated, including carbon-based nanomaterials, metal-based nanomaterials, liposomal formulations, cubosomes, lipid nanoparticles, polymeric nanoparticles, micelles, virus-like and virus-based nanomaterials, exosomes, and even cell membrane-coated nanoconstructs. These nanosized particles may act as effective and efficient vehicles for a plethora of chemotherapeutic drugs, nucleic acids, imaging moieties, photosensitizers, photothermal agents, and other biomolecules. They can be tailored towards delivering their cargo at the tumor site through various surface functionalizations, TME-response mechanisms, or inherent targeting abilities. Thus, the use of tumor-targeting nanoparticles results in higher drug accumulation at the desired site, cancer cell penetration, and enhanced therapeutic activity, leading to higher cytotoxicity against tumor tissues while keeping toxicity in healthy tissues at a minimum.

**Figure 15:**
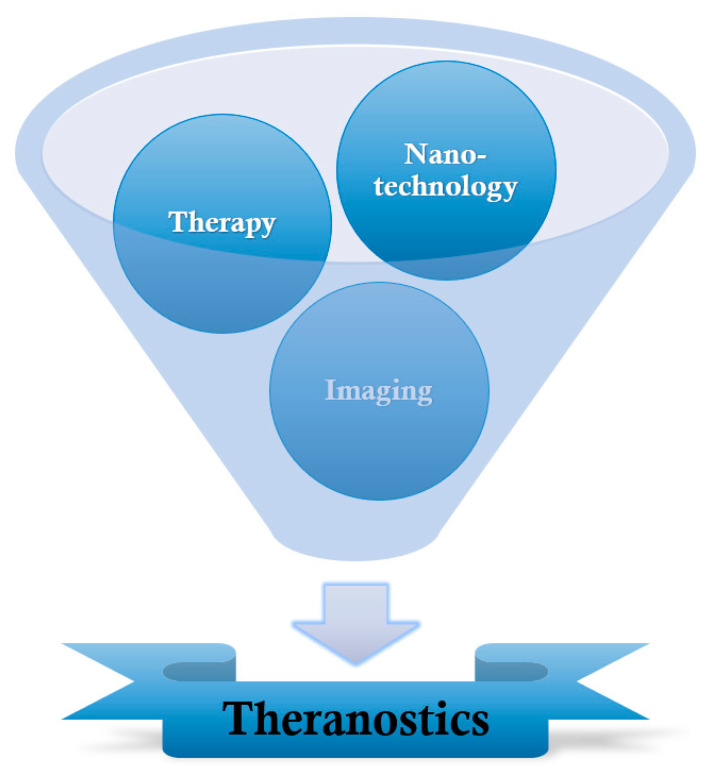
Schematic representation of theranostics.

Moreover, interesting possibilities arise from the ingenious combination of different functionalities within a single nanoconstruct, leading to the emergence of promising theranostic platforms (Figure 15). Hence, numerous studies have evaluated the potential of certain materials for loading and co-delivering drugs, imaging moieties and genes, and identifying tumor cells by binding to specific receptors. Furthermore, such nanosystems have the capacity to generate synergistic outcomes, combining imaging modalities with one or more therapeutic strategies (e.g., chemotherapy, photodynamic therapy, photothermal therapy, radiotherapy, immunotherapy).

To conclude, tumor-targeting nanoparticles represent a highly investigated topic whose implementation in practice would represent a paradigm shift in treating cancer, simultaneously allowing more aggressive and more specific treatments. However, before moving to the clinic, further research must be conducted to elucidate the efficacy and safety of the developed nanosystems for human use, as most of the current studies have only reached the in vitro and in vivo testing stages.

## Figures and Tables

**Table 1:** Summary of adjuvant therapies used in various types of cancer.

Adjuvant Therapy	Advantages	Disadvantages	Type(s) of Cancer	Refs.
Immunotherapy	High accuracy, specificity, and targeting Significantly improves long-term survival rate for “immunoinflammatory” tumor patients Prevents tumor recurrence and metastasis Effective for a long time Fewer side effects than conventional therapies	High treatment costs Poor effect on “immune suppression type” and “immune exclusion type” tumors The use of immune checkpoint inhibitors may produce negative regulation, leading to autoimmune diseases High inter-patient variability Non-specific toxic and side effects may occur in some patients, even leading to disease hyper-progression and accelerated death	Bladder cancer Breast cancer Hodgkin’s lymphoma Lymphatic cancer Melanoma Non-small cell lung cancer Ovarian cancer Renal cancer	[22,23,24]
Hormone therapy	Improved prognoses compared to patients that did not benefit from hormone therapy	Important side effects, including cognitive implications	Breast cancer Endometrial cancer Ovarian cancer Prostate cancer Thyroid cancer	[25,26,27]
Photothermal therapy	Spatiotemporal selectivity Non-invasive Low systemic toxicity High tumor ablation efficiency Slight or no side effects	Treatment efficacy depends on accurate light delivery to the tumor Photothermal absorption is highly dependent on the photothermal transducer Challenging heat confinement	Breast cancer Colorectal cancer Head and neck cancer Pancreatic cancer Skin cancers Thyroid cancer	[28,29,30,31]
Photodynamic therapy	Spatiotemporal selectivity Little invasiveness Minimization of systemic toxicity and minimal functional disturbances Well-tolerated by patients Preserves fertility It can be applied at the same location several times Lower costs compared to other treatment options	Complex scheduling Photosensitivity after treatment Classic photosensitizers limit its application Treatment efficacy depends on accurate light delivery to the tumor Tissue oxygenation is essential in creating the photodynamic effect Not applicable yet to metastatic cancers	Bladder cancer Brain cancer Breast cancer Cervical cancer Colorectal cancer Esophageal cancer Gastric cancer Liver cancer Lung cancer Pancreatic cancer Prostate cancer Skin cancers	[31,32,33,34]
Cryoablation	Successful for local control in various cancer types Superior to other techniques in its ability to preserve native antigen structures Intracellular contents of the damaged tumor cells are preserved and can be recognized by the immune system initiating a tumor-specific immune response	Considerable number of complications (e.g., peripheral bone necrosis, cold injury to surrounding soft tissues) Technically complex procedure Expensive gas-delivery cryoablation systems	Bone tumors Breast cancer Liver cancer Lung cancer Prostate cancer Renal cancer Skin cancers	[24,35,36]
Laser ablation	Minimally invasive alternative to surgery Allows guiding through a flexible and small-fiber to target deep-lying organs Predictable size of necrosis	Not suitable for large tumors	Bladder cancer Breast cancer Colorectal cancer Glioblastoma Liver cancer Lung cancer Osteoid osteoma Pancreas neuroendocrine tumors Penile cancer Prostate cancer Renal cancer	[37]
Radiofrequency ablation	Minimally invasive technique Real-time monitoring of the ablation zone No need for grounding pads Diminished tissue carbonization	Ablation rate decreases with the increase in tumor size Heat sink effect Procedure-related pain	Adrenal glands tumors Bone tumors Breast cancer Hepatocellular carcinoma Lung cancer Pancreatic cancer Renal cancer Thyroid cancer	[38,39,40]
Microwave ablation	Faster ablation speed than RFA Less susceptible to heat sink effect than RFA Less susceptible to tissue impedance than RFA No need for grounding pads No contraindication for patients with metallic implants	Less distinct ablation zone margin than RFA and cryoablation Potential overheating due to rapid energy delivery	Adrenal glands tumors Bone tumors Hepatocellular carcinoma Lung cancer Pancreatic cancer Renal cancer	[38,40,41]
High intensity focused ultrasound	Totally noninvasive Real-time monitoring of thermal effect Immediate assessment of treatment 3D visualization of treatment planning Alleviation of pain and fatigue Overall improvement of quality of life	Extended procedure time for large tumors Imaging artifact and inhomogeneous beam attenuation resulting from thermal protection needles	Bone tumors Brain tumors Breast cancer Liver cancer Parathyroid tumors Pancreatic cancer Prostate cancer Renal cancer Thyroid tumors	[38,42,43]
